# Voxel-wise Fusion of Resting fMRI Networks and Gray Matter Volume for Alzheimer’s Disease Classification using Deep Multimodal Learning

**DOI:** 10.21203/rs.3.rs-3740218/v1

**Published:** 2023-12-13

**Authors:** Vaibhavi S. Itkyal, Anees Abrol, Theodore J. LaGrow, Alex Fedorov, Vince D. Calhoun

**Affiliations:** Emory University; Georgia State University; Georgia Institute of Technology; Emory University; Georgia State University

## Abstract

Alzheimer’s disease (AD) is a prevalent neurodegenerative disorder requiring accurate and early diagnosis for effective treatment. Resting-state functional magnetic resonance imaging (rs-fMRI) and gray matter volume analysis from structural MRI have emerged as valuable tools for investigating AD-related brain alterations. However, the potential benefits of integrating these modalities using deep learning techniques remain unexplored. In this study, we propose a novel framework that fuses composite images of multiple rs-fMRI networks (called voxelwise intensity projection) and gray matter segmentation images through a deep learning approach for improved AD classification. We demonstrate the superiority of fMRI networks over commonly used metrics such as amplitude of low-frequency fluctuations (ALFF) and fractional ALFF in capturing spatial maps critical for AD classification. We use a multi-channel convolutional neural network incorporating the AlexNet dropout architecture to effectively model spatial and temporal dependencies in the integrated data. Extensive experiments on the Alzheimer’s Disease Neuroimaging Initiative (ADNI) dataset of AD patients and cognitively normal (CN) validate the efficacy of our approach, showcasing improved classification performance of 94.12% test accuracy and an area under the curve (AUC) score of 97.79 compared to existing methods. Our results show that the fusion results generally outperformed the unimodal results. The saliency visualizations also show significant differences in the hippocampus, amygdala, putamen, caudate nucleus, and regions of basal ganglia which are in line with the previous neurobiological literature. Our research offers a novel method to enhance our grasp of AD pathology. By integrating data from various functional networks with structural MRI insights, we significantly improve diagnostic accuracy. This accuracy is further boosted by the effective visualization of this combined information. This lays the groundwork for further studies focused on providing a more accurate and personalized approach to AD diagnosis. The proposed framework and insights gained from fMRI networks provide a promising avenue for future research in deep multimodal fusion and neuroimaging analysis.

## Introduction

There is a dynamic loop between brain structure and brain function which comprises the neural basis of cognition, learning and plasticity (V. Calhoun, 2018; Segall et al., 2012; Sui et al., 2014; Zatorre et al., 2012). As individuals undergo the natural process of aging, the human brain experiences alterations in structure and cognitive capabilities. Notably, rapid transformations in both structure and function, accompanied by symptoms such as dementia, cognitive decline, and the presence of biomarkers such as tau and beta-amyloid deposition, signify the onset of Alzheimer’s Disease (AD) (Beason-Held et al., 2013; Hersi et al., 2017). However, relying solely on tau and β-amyloid levels for predictions related to AD or cognitive outcomes is insufficient for accurate diagnosis and prognosis (Khoury & Ghossoub, 2019), (Hardy & Higgins, 1992; J. C. Lee et al., 2019). The degeneration of the brain associated with Alzheimer’s disease commences years before the manifestation of clinical symptoms. Recent advancements in positron emission tomography (PET) used to visualize the deposition of amyloid and tau have enhanced our ability to detect Alzheimer’s disease during its preclinical and prodromal stages. Issues arise as this approach is encumbered by high costs, ionizing radiation, and necessitates specialized tracers and equipment (Hansson et al., 2019). Given that Alzheimer’s disease affects a significant portion of the global population, early and precise AD diagnosis is imperative to facilitate timely interventions and enhance patient well-being (Cummings et al., 2022; Dubois et al., 2021; L.-K. Huang et al., 2020). To develop effective and clinically relevant early detection methodologies, it is vital to distinguish prodromal AD, which is often studied through the lens of mild cognitive impairment (MCI) (Liss et al., 2021; Sabbagh et al., 2020; Wee et al., 2012). MCI is characterized by individuals affected by dementia with high propensity to progress to more severe forms of dementia. The Alzheimer’s Disease Neuroimaging Initiative (ADNI) has generated and made publicly available a wide array of data, including imaging, clinical, genetic, and various biomarkers, from individuals categorized as being cognitively normal (CN), MCI and AD. ADNI is a multisite study which uses standardized protocols and quality control measures to minimize variations across sites and enhance the reliability of neuroanatomical change detection (Jack Jr. et al., 2008). The study collected both structural magnetic resonance imaging (sMRI) and resting-state functional magnetic resonance imaging (rs-fMRI) data, however most prior work has focused on sMRI and there is much less work focused on joint analysis of sMRI and fMRI data. Ergo, there is untapped potential for harnessing information provided by both sMRI and rs-fMRI for the purpose of differentiating AD, CN and MCI.

Structural imaging techniques, exemplified by the analysis of voxelwise gray matter volume, have showcased their efficacy in identifying AD-related anatomical modifications (Dukart et al., 2013). In parallel, rs-fMRI has been studied as a way to elucidate the functional brain changes associated with AD (Agosta et al., 2012). Prior studies have focused on features such as amplitude of low-frequency fluctuation (ALFF) and fractional amplitude of low-frequency fluctuation (fALFF) to capture spatial patterns within rs-fMRI data (Abrol, Hassanzadeh, et al., 2021; Fedorov et al., 2021), (Turner et al., 2013). ALFF, while useful, has the drawback that it collapses all the information into a spectral power band, thus losing information about widely studied brain networks though some information is still retained (V. D. Calhoun & Allen, 2013) and it can be more sensitive to artifacts that impact the subcortical and periventricular regions disproportionately (Zuo et al., 2010). Overall, structural measures tend to show more sensitivity to AD related changes and are much more widely used as a result. However, no work to our knowledge has evaluated the use of input feature which preserve resting brain networks at the voxelwise level.

In this work we explore novel avenues for representing and synthesizing spatial information from intrinsic networks extractive from rs-fMRI datasets and combining it with gray matter maps. The progression of AI-driven solutions within clinical neuroimaging and the broader healthcare landscape provides the promise of more flexible and powerful models. Despite the emergence of innovative models, most studies have overlooked the integration of multimodal and data fusion techniques (V. D. Calhoun & Sui, 2016; Sui et al., 2012, 2014). Furthermore, investigations into functional dynamics, particularly concerning rs-fMRI datasets, for the comprehension and representation of spatial information, have been limited (for a comprehensive review please see the following(Hutchison et al., 2013; Keilholz et al., 2017; Lurie et al., n.d.)). This gap signifies missed opportunities on multiple fronts: (i) Converting and summarizing functional insights from rs-fMRI data into structural maps, and (ii) Employing deep multimodal neural architectures for the classification of AD, MCI, and CN subjects, along with the utilization of interpretability methods to discern influential features that impact model predictions.

In this study, we introduce an innovative deep learning framework based on a 3D convolutional neural network (CNN) architecture, drawing inspiration from the well-established AlexNet (Abrol, Fu, et al., 2021). Our model exhibits the capability to effectively differentiate between AD, MCI and CN subjects, employing the ADNI dataset (total subjects = 730). Our methodology is designed to harness complementary insights from both modalities: it exploits functional connectivity patterns derived from rs-fMRI and anatomical characteristics obtained through the analysis of gray matter volume. Through voxelwise fusion of these modalities, we reinforce the discriminative capacity of our classification model, consequently enhancing the accuracy of AD diagnosis. Our approach hinges on voxel-wise data fusion of rs-fMRI networks and gray matter volume, facilitated by multimodal deep learning. Rigorous cross-validation experiments substantiate our method’s performance is favorable against existing techniques. Our proposed approach attains an impressive test accuracy of 94.12% when distinguishing between AD and CN within the ADNI dataset (total subjects = 466). Notably, our deep learning model exhibits high accuracy and robustness across multiple experimental scenarios. One of main areas of study in fMRI is focused on resting networks, each of which produced a spatial map of connectivity (functional coupling) at each voxel. However most approaches do not leverage these multiple networks. Here, we introduce a way to incorporate the most important information across a large number of resting networks via creating voxel-wise intensity projection (VIP) networks, generated via independent component analysis (ICA) i.e., ICA followed by VIP i.e., iVIP, compared to conventional metrics such as ALFF and fALFF for AD classification. Moreover, our results showcase the enriched and informative spatial patterns offered by the representation of our fMRI networks, facilitating the precise localization of AD-related alterations. Our findings underscore the substantial promise of CNN-based models for automating early Alzheimer’s disease diagnosis across various stages, signifying the potential to deliver valuable insights into the intricate neurobiological mechanisms underpinning AD. This, in turn, may contribute to early and accurate diagnosis, and thus pave the way for personalized treatment strategies.

## Methods

**Figure 1:**
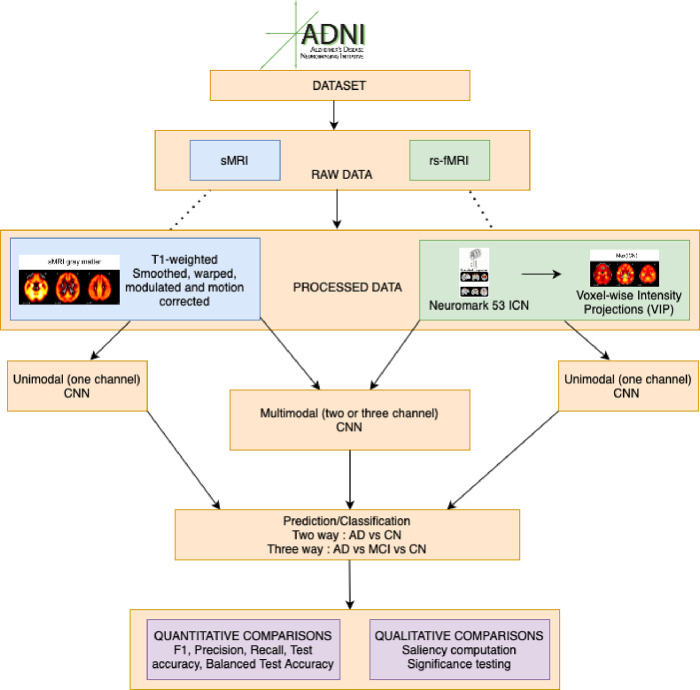
An overview of the data processing and prediction pipeline for the unimodal and multimodal analysis that was conducted in our study. We used the sMRI and rs-fMRI from the ADNI dataset and preprocessed sMRI using SPM 12 and used GIFT toolbox for computing ICNs. After that we computed iVIPs from the ICNs and used them as a different modality with sMRI for our classification predictions. We did a two-way (AD vs CN) and three-way classification (AD vs MCI vs CN). We then compared our results quantitatively as well as using saliency maps for all the experiments.

Previous studies (Egorova et al., 2017; Reyes et al., 2016; Turner et al., 2013 -j. Wang et al., 2016; Zou et al., 2008) have used measures such as ALFF and fALFF for multimodal deep learning. In our paper, we present a new technique named iVIP. This method calculates a voxel-by-voxel summary of resting fMRI networks, similar to those derived through Independent Component Analysis (ICA). We then explore the potential for using iVIP for classification purposes in the ADNI data, and compare sMRI only, fMRI only, and fused fMRI and sMRI scenarios. To do this we utilize a modified version of the 3D CNN AlexNet3D dropout architecture (Krizhevsky et al., 2017). We apply our approach to the ADNI dataset. We perform a two-way (AD vs CN) and three-way (AD vs MCI vs CN) classification task and several experiments to fuse the sMRI and iVIP modalities in our deep learning architecture. In addition, to visualize and determine the relevant brain regions that the network uses for classification, we compute saliency maps from the deep learning model and investigate voxelwise differences between classes. Figure 1 depicts an overview of the proposed approach for performing multimodal deep learning classification using the ADNI dataset.

### Data

The data utilized in this study comprises imaging and diagnostic information sourced from the Alzheimer’s Disease Neuroimaging Initiative (ADNI). The research protocols of the ADNI study were subject to approval by the institutional review boards at all participating centers, and this approval process is meticulously detailed in the official document, which can be accessed via this link: ADNI Approval Documentation.

Prior to inclusion in the study, all individuals provided written, informed consent in full compliance with the ethical principles set forth in the Declaration of Helsinki. Furthermore, this study received explicit approval from the institutional review boards at each research site that was involved in the investigation.

It is imperative to note that all research methodologies adhered rigorously to the established guidelines and ethical standards. The sMRI scans (specifically T1 MRIs) and rs-fMRI scans were procured from the ADNI data portal (https://adni.loni.usc.edu/). For the purposes of this study, sMRI and rs-fMRI data obtained from the ADNI dataset were exclusively derived from the initial visit scans, encompassing a total of 730 subjects. This cohort consisted of 383 individuals classified as CN, 83 subjects diagnosed with AD, and 264 individuals with MCI. The Table 1 summarizes the ADNI data used for two-way vs three-way classification as well as notes the ratio of male and female (M/F) participants used in the study.

### Data Preprocessing

In this study, we utilized sMRI and rs-fMRI data from the ADNI dataset. We focused on the initial visit scans of subjects for whom sMRI, rs-fMRI, and diagnosis or research group information were available and utilized for the subsequent analysis. Data were first preprocessed as in prior work (Abrol et al., 2020), briefly summarized below.

#### sMRI preprocessing:

The preprocessing of the sMRI data commenced with spatial normalization and segmentation of tissue probability maps. This segmentation specifically targeted gray matter, white matter, and cerebral spinal fluid, employing the modulated normalization algorithm within SPM 12. Subsequently, the gray matter images underwent transformation to a standardized space, modulation, and were subjected to smoothing using a Gaussian kernel featuring a full width at half maximum (FWHM) of 6 mm. The resultant preprocessed gray matter volume images were characterized by voxel dimensions of 121 × 145 × 121 and a voxel size of 1.5 × 1.5 × 1.5 mm³ in spatial terms.

To ensure data quality, we conducted a thorough quality control (QC) assessment of the preprocessed sMRI and fMRI datasets. This included the exclusion of images exhibiting low correlation with individual and/or group level masks. For the fMRI data, images manifesting high head motion were meticulously discarded to eliminate potential confounding effects on functional connectivity.

#### fMRI preprocessing:

The preprocessing of the fMRI data was executed through an SPM12 pipeline encompassing rigid body motion correction, slice-timing correction, warping to the standard MNI space employing the EPI template, resampling to isotropic voxels of (3mm)³, and subsequent Gaussian smoothing with a FWHM of 6mm. Leveraging fully automated spatially constrained ICA (scICA) with the Neuromark_fMRI_1.0 template (Du et al., 2020), we obtained 53 spatially independent components characterized by a high correlation threshold for multi-dataset alignment. These components were pre-computed and made accessible via the GIFT toolbox (https://trendscenter.org/software/gift/).

All maps, encompassing sMRI and rs-fMRI processed data, were transformed to the standard MNI space. Subsequently, they were resampled to isotropic voxels of (3mm)³, yielding a consistent voxel dimension of 53 × 63 × 52 voxels within each map. Gaussian smoothing, featuring a FWHM of 6mm, was applied as the final step in the data preparation pipeline.

We estimated 53 intrinsically connected networks (ICNs) via the scICA Neuromark_fMRI_1.0 template followed by computation of the iVIPs. The ICNs are subject specific multi-network ICA networks reflecting synchronized activity (Du et al., 2020). To extract the most important features of the data, we computed the maximum value across the 53 ICNs for each voxel, representing it as max(ICN). Additionally, we computed both the absolute minimum and maximum of the absolute values of the 53 ICNs for each voxel, denoted as abs(min(ICN)) and max(abs(ICN)), respectively. These post-processed 3D iVIP images were employed as input, either unimodally or multimodally, in our neural network model.

In order to benchmark our novel representation of rs-fMRI spatial maps, we conducted a comparative analysis with the widely used ALFF and fALFF approaches, using the preprocessing approach from this previous work (Abrol, Hassanzadeh, et al., 2021). These traditional metrics have been recognized for their data reduction capabilities while preserving voxel dimensionality (Abrol, Hassanzadeh, et al., 2021; V. Calhoun, 2018; Turner et al., 2013).

### Multimodal Deep Convolution Neural Network

A multi-channel version of a 3D CNN inspired from AlexNet (Krizhevsky et al., 2017) was used for classification tasks. Our model is a specialized deep learning architecture tailored for 3D multimodal deep learning which has been designed to address the heightened challenges posed by the curse of dimensionality prevalent in neuroimaging datasets (Alzubaidi et al., 2021; Singh et al., 2020). This model features a sequence of 3D convolutional layers designed to adapt to diverse data modalities, batch normalization for training stability, rectified linear units (ReLU) for nonlinear feature extraction, and adaptive average pooling for spatial adaptability. We have also incorporated dropout layers to prevent overfitting. The model takes three-dimensional data, fMRI or sMRI images, as input, represented as a sequence of 3D volumes. The convolutional layers capture spatial patterns and features in the input volumes at different levels of abstraction. The model progressively reduces the spatial dimensions of the input through max pooling operations. Dropout regularization is applied to mitigate overfitting (Srivastava et al., 2014). The output of the convolutional layers is flattened and passed through a fully connected classifier. The classifier consists of two fully connected layers with ReLU activation and dropout, followed by a final linear layer that produces the model’s predictions. The significance of this model lies in its remarkable aptitude for both two-way (AD vs CN) and three-way classification tasks (AD vs MCI vs CN). Moreover, the final layers of the model include fully connected layers, allowing for the translation of feature representations into classification or regression predictions. The architecture can be customized to accommodate the number of modalities, classes, and channels.

### Experiments

For unimodal and multimodal deep learning, we performed three experiments.

#### Experiment 1:

**Figure 2:**
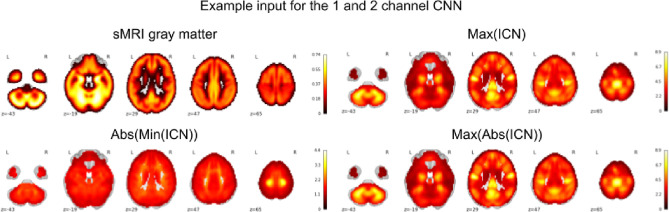
Processed sMRI and rs-fMRI which were used as the inputs for the 1 and 2 channel CNN in this study. This is a montage representation of the inputs that were used in Experiment 1 and Experiment 2. Note that the iVIP is a general/umbrella term for max(ICN), abs(min(ICN)) or max(abs(ICN)).

We used a single channel CNN with one modality at a time – i.e., sMRI or max(ICN) or abs(min(ICN)) or max(abs(ICN)). We also did a unimodal comparison with the existing state-of-the art rs-fMRI spatial map representation (such as ALFF and fALFF) with the iVIP. The inputs for the 1 channel CNN are visualized in Fig. 2.

#### Experiment 2:

We used a two-channel CNN with one modality (iVIP or sMRI) in each channel. Thus, we had channel 1 as the sMRI and channel 2 included either max(ICN) or abs(min(ICN)) or max(abs(ICN)). The different inputs for the 2 channel CNN are depicted in Fig. 2.

#### Experiment 3:

**Figure 4:**
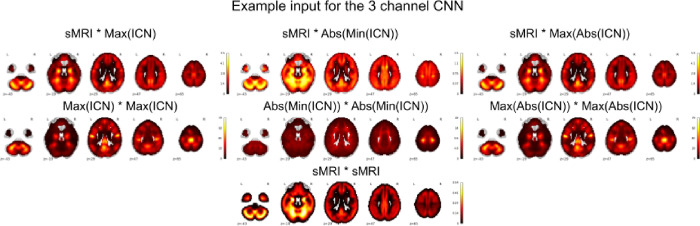
Inputs for 3 channel 3D CNN. These are the different inputs which were used as different channels in the 3 channel CNN. We used sMRI*sMRI as channel 1, sMRI*iVIP as channel 2 and iVIP*iVIP as the channel 3. Note iVIP varied in each case between max(ICN), abs(min(ICN)) or max(abs(ICN)).

We used a three channel CNN with fused modalities. Channel 1 was the square of sMRI, channel 2 was the product of the two modalities i.e., sMRI * iVIP whereas channel 3 was the square of the iVIP modality. Note here the iVIP modality was either max(ICN), abs(min(ICN)) or max(abs(ICN)). The inputs in our different 3 channel CNN are visualized in Fig. 4.

### DL training

We conducted training and testing procedures for the deep learning architectures on an NVIDIA CUDA parallel computing platform. This platform utilized two Intel(R) Xeon(R) Gold 6230 CPUs operating at 2.10 GHz, and it was hosted on the TReNDS slurm-managed cluster. This cluster featured nodes equipped with 4 NVIDIA Tesla V100 SXM2 32 GB GPUs, enabling efficient parallel processing. We leveraged the GPU-accelerated NVIDIA CUDA toolkit (cudatoolkit), CUDA Deep Neural Network (cudnn), and PyTorch tensor libraries to enhance computational performance. For optimizing the models, we adopted the Adam algorithm, as implemented in the torch.optim package. This choice was motivated by its computational efficiency, minimal memory requirements, and suitability for tasks involving high-dimensional parameter spaces. In our custom code, we also made use of several essential packages, including nipy, scipy, numpy, nibabel, and pandas. These packages played crucial roles in basic image processing and read-write operations. In all experiments, a learning rate scheduler callback was employed. It dynamically reduced the learning rate by a factor of 0.5 when the validation accuracy metric reached a plateau. To mitigate overfitting and enhance generalization performance in the testing phase, early stopping was implemented. It had a patience level of 20 epochs for the classification tasks and 40 epochs for the regression tasks. Cross-entropy loss function was used for classification. To ensure a fair and consistent comparison of time complexity in the deep learning runs, each run was allocated a maximum of 8 CPU threads.

We performed 8-fold cross-validation (CV) for each experiment. Splitting of the train and test set was done separately for two-way and three-way classification using the stratified Monte Carlo i.e., randomized and repetitive sub-sampling CV. The data for the binary classification (AD vs CN) was partitioned into training (350 scans), validation (58 scans), and test sets (58 scans). For the three-way classification (AD vs MCI vs CN), 540 scans were allocated to the training set, while both the test and validation cohorts received 95 scans each. We also ensured stratification of the data during train and test splitting based on the two (AD vs CN) and three (AD vs MCI vs CN) classes respectively. For each iteration, the training, validation, and test samples were selected exactly once, ensuring consistency in the comparison by maintaining their uniformity across various experiments. In each repetition, hyperparameter tuning was conducted using the validation dataset, and the reported performance metrics were assessed using the independent held-out test set. A mask was computed for each subject with a threshold of sMRI voxels > 0.03. This mask was used for the same subject’s sMRI as well as iVIP. After masking we applied min-max normalization to ensure that each CNN channel has values between [0,1]. Hence the major difference between the maps is that the CN have a higher maximum value than the AD. For the Experiment 3 where we computed product, square; we ensured that the min-max normalization was done (as it ensures equal distribution of the modalities in the result) after the computation of each modality as well as after computing the product and square of the fused modalities. All the three experiments were computed separately for two (AD vs CN) and three (AD vs MCI vs CN) way classification.

### Hyperparameters

Based on prior work (Abrol, Fu, et al., 2021), We performed an exhaustive hyperparameter tuning for different learning rate (0.1, 0.01, 0.001, 0.0001 and 0.00001), batch sizes (4, 8, 16, 32, 64) as well as combination of kernel size-stride length-padding (5–2-0 or 3–2-1). We tuned values for each experiment and reported the quantitative results for the optimized hyperparameters for each experiment.

### Saliency and voxel-wise statistical analysis

Saliency maps were generated for individual test subjects through the application of guided back-propagation (Simonyan et al., 2014) on the trained neural network architecture. In the context of multimodal architectures, it’s essential to emphasize that these saliency maps are computed separately for each input feature map. To facilitate a comparative analysis of saliency the maps derived from all the subjects in each experiment were smoothed with a FWHM = 10 and then averaged. This procedure was carried out for both two-way and three-way classification.

## Results

Our study leveraged sMRI and rs-fMRI data from the ADNI dataset each associated with one of three cognitive status categories: AD or MCI or CN. To facilitate classification, two distinct tasks were undertaken: a binary classification (AD vs. CN) and a ternary classification (AD vs. MCI vs. CN) using our 3D CNN architecture. A total of 466 subjects were used for the binary classification. For the three-way classification, a total of 730 subjects were used. Importantly, only a single scan per subject was utilized in both binary and ternary classification analyses. Please refer to Table 1 for more details about the data. The study employed a specialized 3D CNN model tailored for multiclass classification, designed to excel in distinguishing AD, MCI or CN statuses based on sMRI and fMRI data, serving as the primary tool for predictive performance and subsequent analyses.

### ALFF/fALFF vs iVIP

Our proposed approach utilized a novel feature called iVIP which used intensity projection to create a voxelwise summary of 53 spatial ICN networks using max(ICN) or abs(min(ICN)) or max(abs(ICN)) was compared with the widely used ALFF/fALFF methods. Table 2 shows the test accuracy as well as balanced test accuracy comparison over an 8-fold cross validation as a part of the Experiment 1. We used a repeated stratified sub-sampling procedure for these unimodal comparisons. The Table 2 clearly indicates that the iVIP outperforms the ALFF and fALFF measures quantitatively. We note these findings, particularly from the results of the two-way classification that the performance of the iVIP (max(ICN) or abs(min(ICN)) or max(abs(ICN))), is higher than the corresponding ALFF/fALFF results. The same 3D CNN architecture was used for the one channel classification and the hyperparameters were tuned separated for each modality (iVIP or fALFF or ALFF).

Results (Table 2) also showed that the DL models from iVIP outperform the corresponding ALFF/fALFF models with the highest test accuracy of 85.02% by max(abs(ICN)) for the AD vs CN classification and 51.38% by max(ICN) for the AD vs MCI vs CN classification. We also note that the AUC value for two-way and three-way classification is higher for all the iVIPs than the corresponding ALFF/fALFF AUC. The highest AUC for two-way classification was noted by the max(ICN) at a value of 83.58 whereas for the three-way classification we observe that max(abs(ICN)) with a value of 60.96 outperforms the other modalities. A chi-squared test on the test accuracies showed, for all different fMRI measures, iVIP was significantly higher than ALFF/fALFF (iVIP vs p-ALFF = 0.000008 and iVIP vs p-fALFF = 0.000001). This was also the case for the max(ICN) vs ALFF/fALFF (p = 0.000048) as well as for abs(min(ICN)) vs ALFF/fALFF (p < 0.000006). For the three-way classification i.e., AD vs MCI vs CN, iVIP (only max(ICN) and max(abs(ICN))) also significantly outperforms fALFF/ALFF measures (p = 0.000001). Whereas on performing the chi-squared test on the AUC values for the iVIP vs respective fALFF/ALFF values, we determined iVIP outperformed ALFF/fALFF measures.

Based on our quantitative comparisons we find that the proposed iVIP measures sigificantly outperforms ALFF/fALFF for two-way and three-way classification. iVIP’s test and balanced accuracy scores are higher or similar to ALFF’s in some cases but overall, we report a significant difference in the AUC values. Thus, we used iVIP measures in our further multimodal analysis of Experiment 2 and Experiment 3.

### Quantitative analysis: performance comparison

We performed two-way and three-way classification for Experiment 2 and 3. iVIP was used in the multimodal models which had sMRI and rs-fMRI data. We used a single channel 3D CNN network for Experiment 1, and 2-channel and 3-channel 3D CNN networks for Experiment 2 and 3, respectively. Our Experiment 2 results are summarized under the rows labeled ‘sMRI + iVIP’ whereas our Experiment 3 results are summarized under the rows labeled as ‘Fused’. Tables 3 and 4 summarizes our findings for two-way and three-way classification results respectively.

Results show unimodal sMRI outperforms unimodal iVIP, and, importantly, the fused models outperform both unimodal models. That is, sMRI + iVIP as well as the fused multimodal model (sMRI*sMRI + sMRI * iVIP + iVIP * iVIP) perform the best. The highest performing test accuracy (94.12%) for the two-way classification is from the fused model with abs(min(ICN)). The fused model with abs(min(ICN)) test accuracy is also significantly higher than the corresponding unimodal measures (p < 0.05).

Our results from Table 3 also show that the fused models are significantly better (p < 0.05) than the corresponding unimodal models for two-way classification in all cases. We also note that the AUC value of 97.79, is the highest for the fused model of the abs(min(ICN)).

Similar to Table 3, we observe that the unimodal sMRI performs better than the unimodal iVIP, but we see a significant increase in the results from the multimodal models i.e., sMRI + iVIP and the fused multimodal model (sMRI*sMRI + sMRI * iVIP + iVIP * iVIP). The highest performing test accuracy (62.68%) for the three-way classification is from the multimodal model i.e., sMRI + abs(min(ICN)). Statistical significance analysis shows that that for the 730 subjects in the three-way classification, all our Experiment 3 results i.e., the ‘fused’ model, are significantly better than the corresponding unimodal models based on their test accuracy scores (p < 0.05). We also note that all our multimodal model’s AUC scores are significantly higher than the unimodal model’s scores with the highest AUC as 68.31 for the three-way classification using the fused model with the max(abs(ICN)).

### Qualitative Analysis: Visualization of Saliency Maps

**Figure 5:**
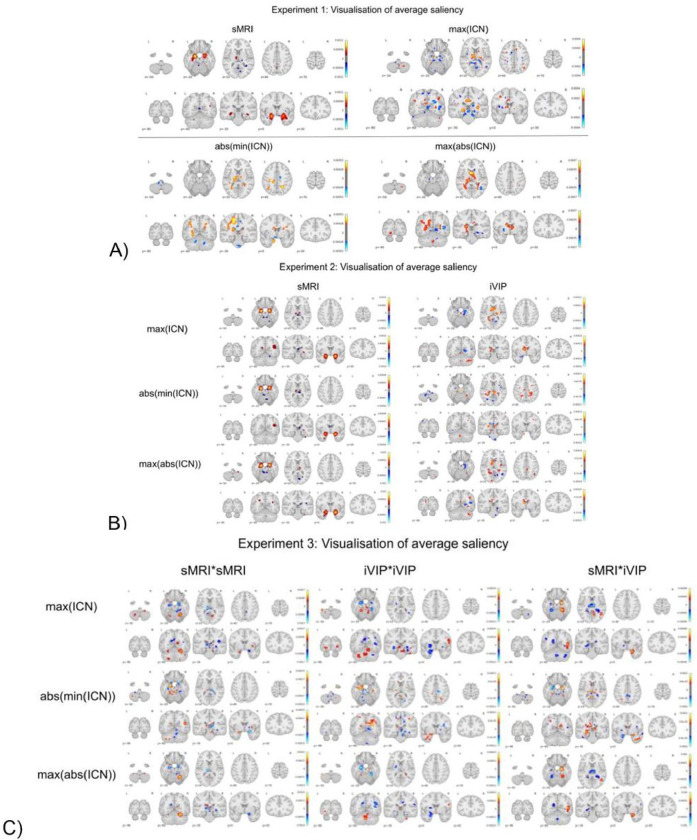
Visualisation of saliency maps for two-way classification (AD vs CN). A) Experiment 1 i.e only one modality (sMRI or max(ICN) or abs(min(ICN)) or max(abs(ICN))) was used at a time for predicting the classes. B) Experiment 2 i.e two modalities were used at the same time for predicting the classes via a 2-channel 3D CNN. The first channel was the sMRI modality. The second channel varied for different iVIP. C) Experiment 3 saliency maps i.e from a 3-channel 3D CNN where each channel as sMRI*sMRI or sMRI*iVIP or iVIP*iVIP. Note: All the maps were constructed from the saliency values after masking, smoothing and averaging them.

**Figure 6:**
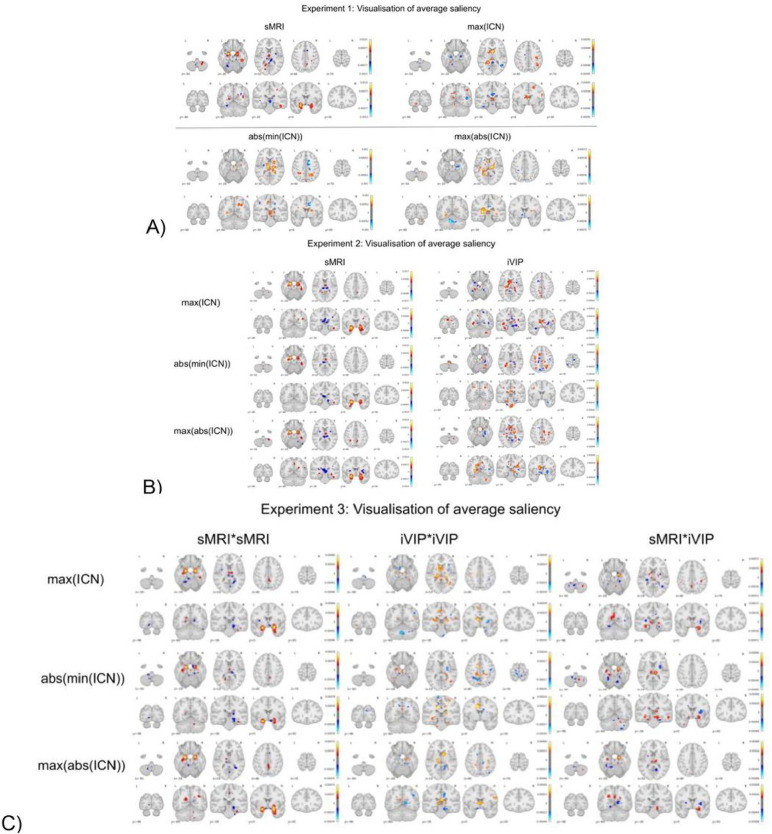
Visualisation of saliency maps for three-way classification (AD vs MCI vs CN). A) In Experiment 1, individual modalities such as sMRI, max(ICN), abs(min(ICN)), or max(abs(ICN)) were utilized independently to predict classes. B) Experiment 2 involved using two modalities simultaneously in a 2-channel 3D CNN. The first channel represented the sMRI modality, while the second channel varied based on different iVIP. C) In Experiment 3, saliency maps were generated using a 3-channel 3D CNN. Each channel represented combinations such as sMRI*sMRI, sMRI*iVIP, or iVIP*iVIP. All saliency maps were obtained after masking, smoothing, and averaging.

We extracted the saliency maps, using guided back-propagation on a trained neural network, for individual test subjects. We visualised these saliency maps after smoothing, averaging and thresholding to enable a comparative analysis in the context of different experiments for both two-way (Fig. 5) and three-way (Fig. 6) classification experiments.

Age-related alterations impact diverse brain systems, influencing memory and cognitive control. Our saliency maps from the two-way classification (Fig. 5) indicate that in Experiment 1 and 2, the sMRI maps show the areas of hippocampus, amygdala, and caudate nucleus. We see that from the visualization of max(ICN) we see major differences in the cingulate cortex, thalamus and caudate nucleus; whereas the abs(min(ICN)) show differences in the thalamus, cingulate cortex and caudate nucleus. The max(abs(ICN)) only shows major differences in the thalamus and caudate nucleus. But for the three-way classification results besides the same regions from the two-way classification for Experiment 1 and 2, we observe additional regions with major differences. For sMRI the cingulate cortex shows variations but major differences in the putamen, caudate nucleus and globus pallidus are observed for the abs(min(ICN)) and max(abs(ICN)) modality. For experiment 3, in two-way classification results we observe variations in the hippocampus, caudate nucleus, subthalamic nucleus, substantia niagra, amygdala, putamen, and thalamus for the three channels (i.e sMRI*sMRI, sMRI*iVIP and iVIP*iVIP). Whereas for the three-way classification results we observe that the major differences are from hippocampus from all the three channels followed by thalamus, amygdala and subthalamic nucleus.

## Discussion

Our research work provides a novel method for the representation of the 4D rs-fMRI dataset in spatial maps which summaries the different functional networks (ICNs) using the iVIP, followed by a unimodal or multimodal deep 3D CNN classifier. The proposed iVIP approach for voxel-wise intensity projections of the ICNs summarizes the spatial information from the 4D rs-fMRI dataset using sc-ICA. After, iVIP the max(ICN), abs(min(ICN)) and max(abs(ICN)) basically were computed to identify the voxels with the most activating ICN, most absent or least activating ICN, most extreme ICN which is regardless of its direction. For two-way as well as three-way classification, the test accuracy as well as AUC scores for iVIP were the highest. Our quantitative results evidently indicated that the iVIP method outperformed previously and more commonly used method for spatial map representation i.e., ALFF and fALFF. Using iVIP we are able to summarize the most important contribution of the resting networks, hence we further used the iVIP representation along with sMRI, unimodally as well as multimodally to classify the research group categories (AD vs CN or AD vs MCI vs CN) from the ADNI dataset.

Most of the best performing models from previous work pertaining image classification using MRI datasets used AlexNet architecture (Abrol, Fu, et al., 2021; Naz et al., 2022; Rahim et al., 2023), hence we used our model which was an adapted version of the AlexNet model. We performed several unimodal as well as multimodal classification experiments with fused modalities as well as multichannel 3D CNN representation to predict the research classes. Quantitative results (e.g., test accuracy scores) show that the fused multimodal model from Experiment 3 performed significantly better than the respective unimodal models for two-way classification (AD vs CN) as well as three-way classification (AD vs MCI vs CN). Our best performing model from the two-way classification had the highest test accuracy of 94.12% and AUC of 97.79 from the fused model. The highest accuracy for the three-way classification results was 62.68% with an AUC of 68.31 from the fused model. Our fused models allow us to incorporate the most critical resting state networks fused with the sMRI. Thus, our results show a high classification accuracy with multimodal models generally outperforming the unimodal models.

Aging affects regions such as the hippocampus, putamen, middle temporal, post-central, superior, and mid-frontal gyri, consistent with prior research on age-related changes in gray matter volume and functional connectivity (Salami et al., 2012). Our saliency results for sMRI indicate major differences in the hippocampus, subthalamic nucleus, amygdala, and caudate nucleus which is in line with the neurobiological literature (de Jong et al., 2008; Heimer, 1983; Selden et al., 1994; Z. Wang et al., 2016). Whereas our iVIP modality shows major differences in the cingulate cortex, subthalamic nucleus, substantia niagra, thalamus, caudate nucleus, putamen, globus pallidus which is also in line with the literature for functional changes in the brain due to AD (Amanzio et al., 2011; Botzung et al., 2019; de Jong et al., 2008; Grahn et al., 2008; Heimer, 1983; C. Huang et al., 2002; P.-L. Lee et al., 2020; Lehéricy et al., 1991; Mattila et al., 2002; Selden et al., 1994). Also, we see that the fused model i.e., Experiment 3 for two-way classification gives insights into more relevant regions such as the subthalamic nucleus, substantia niagra, amygdala, putamen besides the other regions that are relevant for the diagnosis of AD but missed in the unimodal models or the non-fused models. Hence, on examining of saliency results, we demonstrate the potential of deep learning techniques to yield more nuanced and varied interpretations of significant high-dimensional neuroimaging features. In summary, our findings suggest that the patterns of discrimination captured by deep learning models in diagnostic classification align with established neurobiological knowledge regarding the brain regions that experience both structural and functional changes in Alzheimer’s disease.

Thus, our proposed multimodal disease classification framework based on 3D CNN in this study shows the potential for a unified, end-to-end multimodal fusion approach as our multimodal models significantly performed better than the corresponding unimodal models. Our approach is adaptable and could be used to train data, whether it’s raw or preprocessed, and features from all modalities. Our multimodal framework offers improved tuning and optimization of network weights compared to the unimodal training approach employed in the previous studies (Tajammal et al., 2023; Tufail et al., 2020). Nevertheless, our initial findings provide substantial evidence of the advantages of disease classification through multimodal fusion using deep learning, combining sMRI and iVIP spatial maps that summarize rs-fMRI ICN.

Our study has some limitations, firstly that the maximum number of subjects used for analysis was 730, which, while large enough to train the current models and provide some confidence in the results, it does not fully leverage the potential of nonlinear models. Our future research would hence aim at leveraging other AD datasets as well as incorporating multiple scans from the same subject. Secondly, we used voxel-wise saliency maps for visualization which are sometimes sensitive to the hyperparameters of the 3D neural network and the initial parameters of the network. Although our saliency maps were computed for each subject across several folds which would have combatted this limitation, in our future work we will employ multiple other alternative interpretability methods such as SHapley Additive exPlanations (SHAP) (Lundberg & Lee, 2017). A notable constraint in our investigation is the absence of a comparison among various deep learning architectures. Instead, we opted for a comparison with the widely acknowledged and high-performing AlexNet models from comparative literature which demonstrated best results with this model (Abrol, Fu, et al., 2021; Naz et al., 2022; Rahim et al., 2023). This decision was made to mitigate biases arising from diverse architectures and ensure a more robust evaluation of our results with the existing state-of-the-art architecture for the 3D image classification of the AD dataset. This provides us for an opportunity for future research which could encompass a comprehensive comparison of various deep learning architectures to determine the most suitable framework for diagnostic classification and predictive tasks in the realm of neuroimaging data analysis. Additionally, we are keen to explore the progression of AD using longitudinal datasets and expanding the scope by incorporating multiple AD datasets such as the Open Access Series of Imaging Studies (OASIS) (LaMontagne et al., 2019) in our future work. Although, our current study primarily focuses on classification applications, the adaptability of this framework to study and predict important continuous variables such as brain age and clinical assessments based on regression is readily feasible.

## Figures and Tables

**Figure 3:**
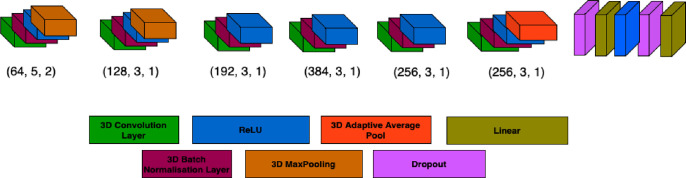
Illustration of the multimodal 3D CNN architecture which we used in our analysis. The structural and functional features from Figure 2 were used as inputs for one channel or multichannel input in the network. The network would predict a class of the research group i.e., AD or CN or MCI depending on whether it was a two-way or three-way classification task.

**Table 1:** Summary of the ADNI data used for classification. 8-fold cross-validation was used for both two-way and three-way classification.

#Subjects	M/F	Total subjects	AD	MCI	CN
Two-way classification (AD vs CN)	188/278	466	83	-	383
Three-way classification (AD vs MCI vs CN)	330/400	730	83	264	383

**Table 2:** Comparing prediction accuracies of our novel representation (iVIP) with the established ALFF and fALFF computations for the rs-fMRI dataset. The values in the table are represented in the format: mean ± standard deviation. The maximum values across different fMRI measures (i.e., for each column) for several accuracy or AUC scores are bold.

	Two-way classification (AD vs CN)			Three-way classification (AD vs MCI vs CN)		
Different fMRI measures	Test Accuracy	Balanced Test Accuracy	AUC	Test Accuracy	Balanced Test Accuracy	AUC
ALFF	80.25 ± 0.35	64.44 ± 1.6	70.01 ± 0.19	50.01 ± 0.13	36.12 ± 0.21	49.96 ± 0.39
fALFF	80.8 ± 0.5	60.71 ±3.5	71.96 ± 0.3	46.95 ± 0.24	40.82 ± 0.25	52.13 ± 0.01
max(ICN)	84.81 ± 0.2	**71.6 ± 0.95**	**83.58 ± 0.53**	**51.38 ± 0.2**	**40.97 ± 0.15**	59.83 ± 0.04
abs(min(ICN))	84.05 ± 0.18	66.6 ± 0.63	79.69 ± 0.29	49.96 ± 0.21	38.75 ± 0.15	60.26 ± 0.09
max(abs(ICN))	**85.02 ± 0.17**	71.47 ± 0.64	83.4 ± 0.49	50.94 ± 0.17	40.72 ± 0.15	**60.96 ± 0.9**

**Table 3:** Performance for the two-way classification experiments (AD vs CN) with 466 subjects

	Test Accuracy	Balanced Test Accuracy	F1	Precision	Recall	AUC
sMRI	91.18 ± 0.13	86.36 ± 0.69	76.67 ± 1.15	76.31 ± 2.28	81 ± 2.85	96.26 ± 0.09
max(ICN)						
iVIP	84.81 ± 0.2	71.6 ± 0.95	49.91 ± 2.2	53.41 ± 5.28	52.3 ± 3.99	83.58 ± 0.53
sMRI + iVIP	91.88 ± 0.18	84.28 ± 0.9	76.37 ± 1.6	77.28 ± 2.62	80.23 ± 3.6	96.88 ± 0.08
Fused	92.38 ± 0.18	86.09 ± 0.64	77.74 ± 1.54	74.37 ± 3.15	81.61 ± 2.34	96.9 ± 0.18
abs(min(ICN))						
iVIP	84.05 ± 0.18	66.6 ± 0.63	42.22 ± 2.5	48.71 ± 5.2	41 ± 2.8	79.69 ± 0.29
sMRI + iVIP	93.31 ± 0.11	**88.43 ± 0.67**	78.52 ± 0.88	79.99 ± 1.5	80.4 ± 2.86	97.11 ± 0.1
Fused	**94.12 ± 0.13**	88.29 ± 0.64	**78.75 ± 1.65**	**81.36 ± 3.4**	**83.63 ± 2.69**	**97.79 ± 0.18**
max(abs(ICN))						
iVIP	85.02 ± 0.17	71.47 ± 0.64	49.34 ± 1.25	54.37 ± 3.45	51.15 ± 3.26	83.4 ± 0.49
sMRI + iVIP	91.81 ± 0.25	83.23 ± 1.03	72.1 ± 1.96	79.53 ± 2.69	69.64 ± 3.85	95.59 ± 0.15
Fused	92.74 ± 0.13	85.06 ± 0.56	74.84 ± 1.24	80.56 ± 2.7	73.47 ± 2.31	95.02 ± 0.2

Note: In Table 3 and 4, ‘Fused’ indicates a combined version of sMRI and iVIP in the following way - sMRI*sMRI + sMRI * iVIP + iVIP * iVIP. Note that different channels in the CNN are separated by a ‘+’ sign. The values in the table are represented in the format: mean ± standard deviation. The maximum values across different models (i.e., for each column) for accuracy, F1, precision, recall, AUC scores are bold.

**Table 4:** Performance for the three-way classification experiments (AD vs MCI vs CN) for 730 subjects

	Test Accuracy	Balanced Test Accuracy	F1	Precision	Recall	AUC
sMRI	58.08 ± 0.09	54.85 ± 0.19	56.70 ± 0.2	63.16 ± 0.33	54.85 ± 0.19	62.26 ± 0.63
max(ICN)						
iVIP	51.38 ± 0.2	40.97 ± 0.15	39.81 ± 0.26	51.51 ± 1.73	40.97 ± 0.16	59.83 ± 0.04
sMRI + iVIP	60.31 ± 0.06	55.39 ± 0.24	56.09 ± 0.2	63.59 ± 0.25	55.39 ± 0.24	66.69 ± 0.11
Fused	60.72 ± 0.19	56.95 ± 0.22	57.70 ± 0.24	64.79 ± 0.36	55.95 ± 0.22	66.96 ± 0.19
abs(min(ICN))						
iVIP	49.96 ± 0.21	38.75 ± 0.15	37.32 ± 0.21	51.31 ± 1.96	38.75 ± 0.15	60.26 ± 0.09
sMRI + iVIP	**62.68 ± 0.13**	58.65 ± 0.32	58.28 ± 0.37	65.36 ± 0.52	58.65 ± 0.33	66.56 ± 0.19
Fused	62.55 ± 0.15	58.78 ± 0.34	59.10 ± 0.43	63.37 ± 0.49	56.78 ± 0.36	67.06 ± 0.21
max(abs(ICN))						
iVIP	50.94 ± 0.17	40.72 ± 0.15	39.56 ± 0.23	50.78 ± 1.63	40.72 ± 0.16	60.96 ± 0.9
sMRI + iVIP	60.5 ± 0.15	51.73 ± 0.21	56.09 ± 0.25	64.71 ± 0.37	55.73 ± 0.21	67.26 ± 0.63
Fused	60.46 ± 0.17	51.55 ± 0.29	57.25 ± 0.32	63.19 ± 0.45	57.56 ± 0.2	**68.31 ± 0.01**

## Data Availability

The datasets used, processed, and analyzed during our study current study are available in the repository, Alzheimer’s Disease Neuroimaging Initiative (ADNI). The sMRI scans (specifically T1 MRIs) and rs-fMRI scans were procured from the ADNI data portal (https://adni.loni.usc.edu/). Data used in the preparation of this article were obtained from the Alzheimer’s Disease Neuroimaging Initiative (ADNI) database (adni.loni.usc.edu). The ADNI was launched in 2003 as a public-private partnership, led by Principal Investigator Michael W. Weiner, MD. The primary goal of ADNI has been to test whether serial magnetic resonance imaging (MRI), positron emission tomography (PET), other biological markers, and clinical and neuropsychological assessment can be combined to measure the progression of mild cognitive impairment (MCI) and early Alzheimer’s disease (AD). For up-to-date information, see www.adni-info.org.
